# Pretreatment CD4 Cell Slope and Progression to AIDS or Death in HIV-Infected Patients Initiating Antiretroviral Therapy—The CASCADE Collaboration: A Collaboration of 23 Cohort Studies

**DOI:** 10.1371/journal.pmed.1000239

**Published:** 2010-02-23

**Authors:** Marcel Wolbers, Abdel Babiker, Caroline Sabin, Jim Young, Maria Dorrucci, Geneviève Chêne, Cristina Mussini, Kholoud Porter, Heiner C. Bucher, on behalf of the CASCADE Collaboration

**Affiliations:** 1 Basel Institute for Clinical Epidemiology and Biostatistics, University Hospital Basel, Basel, Switzerland; 2 Oxford University Clinical Research Unit, Wellcome Trust Major Overseas Program, Hospital for Tropical Diseases, Ho Chi Minh City, Vietnam; 3 Medical Research Council Clinical Trials Unit, London, United Kingdom; 4 University College London Medical School, London, United Kingdom; 5 Istituto Superiore di Sanità, Rome, Italy; 6 Université Bordeaux II, Bordeaux, France; 7 University of Modena and Reggio Emilia and Azienda Policlinico, Modena, Italy; San Francisco General Hospital, United States of America

## Abstract

**Background:**

CD4 cell count is a strong predictor of the subsequent risk of AIDS or death in HIV-infected patients initiating combination antiretroviral therapy (cART). It is not known whether the rate of CD4 cell decline prior to therapy is related to prognosis and should, therefore, influence the decision on when to initiate cART.

**Methods and Findings:**

We carried out survival analyses of patients from the 23 cohorts of the CASCADE (Concerted Action on SeroConversion to AIDS and Death in Europe) collaboration with a known date of HIV seroconversion and with at least two CD4 measurements prior to initiating cART. For each patient, a pre-cART CD4 slope was estimated using a linear mixed effects model. Our primary outcome was time from initiating cART to a first new AIDS event or death. We included 2,820 treatment-naïve patients initiating cART with a median (interquartile range) pre-cART CD4 cell decline of 61 (46–81) cells/µl per year; 255 patients subsequently experienced a new AIDS event or death and 125 patients died. In an analysis adjusted for established risk factors, the hazard ratio for AIDS or death was 1.01 (95% confidence interval 0.97–1.04) for each 10 cells/µl per year reduction in pre-cART CD4 cell decline. There was also no association between pre-cART CD4 cell slope and survival. Alternative estimates of CD4 cell slope gave similar results. In 1,731 AIDS-free patients with >350 CD4 cells/µl from the pre-cART era, the rate of CD4 cell decline was also not significantly associated with progression to AIDS or death (hazard ratio 0.99, 95% confidence interval 0.94–1.03, for each 10 cells/µl per year reduction in CD4 cell decline).

**Conclusions:**

The CD4 cell slope does not improve the prediction of clinical outcome in patients with a CD4 cell count above 350 cells/µl. Knowledge of the current CD4 cell count is sufficient when deciding whether to initiate cART in asymptomatic patients.

*Please see later in the article for the Editors' Summary*

## Abstract 2

Analyzing data from several thousand cohort study participants, Marcel Wolbers and colleagues find that the rate of CD4 T cell decline is not useful in deciding when to start HIV treatment.

## Editors' Summary

**Background:**

More than 30 million people are currently infected with the human immunodeficiency virus (HIV), the cause of acquired immunodeficiency syndrome (AIDS). Most people who become infected with HIV do not become ill immediately although some develop a short flu-like illness shortly after infection. This illness is called “seroconversion” illness because it coincides with the appearance of antibodies to HIV in the blood. The next stage of HIV infection has no major symptoms and may last up to 10 years. During this time, HIV slowly destroys immune system cells (including CD4 cells, a type of lymphocyte). Without treatment, the immune system loses the ability to fight off infections by other disease-causing organisms and HIV-positive people then develop so-called opportunistic infections, Kaposi sarcoma (a skin cancer), or non-Hodgkin lymphoma (a cancer of the lymph nodes) that determine the diagnosis of AIDS. Although HIV-positive people used to die within 10 years of infection on average, the development in 1996 of combination antiretroviral therapy (cART; cocktails of powerful antiretroviral drugs) means that, at least for people living in developed countries, HIV/AIDS is now a chronic, treatable condition.

**Why Was This Study Done?:**

The number of CD4 cells in the blood is a strong predictor of the likelihood of AIDS or death in untreated HIV-positive individuals and in people starting cART. Current guidelines recommend, therefore, that cART is started in HIV-positive patients without symptoms when their CD4 cell count drops below a specified cutoff level (typically 350 cells/µl.) In addition, several guidelines suggest that clinicians should also consider cART in symptom-free HIV-positive patients with a CD4 cell count above the cutoff level if their CD4 cell count has rapidly declined. However, it is not actually known whether the rate of CD4 cell decline (so-called “CD4 slope”) before initiating cART is related to a patient's outcome, so should clinicians consider this measurement when deciding whether to initiate cART? In this study, the researchers use data from CASCADE (Concerted Action on SeroConversion to AIDS and Death in Europe), a large collaborative study of 23 groups of HIV-positive individuals whose approximate date of HIV infection is known, to answer this question.

**What Did the Researchers Do and Find?:**

The researchers undertook survival analyses of patients in the CASCADE collaboration for whom at least two CD4 cell counts had been recorded before starting cART. They calculated a pre-cART CD4 cell count slope from these counts and used statistical methods to investigate whether there was an association between the rate of decline in CD4 cell count and the time from initiating cART to the primary outcome—a first new AIDS-defining event or death. 2820 HIV-positive patients initiating cART were included in the study; the average pre-cART CD4 cell decline among them was 61 cells/µl/year. 255 of the patients experienced a new AIDS-related event or died after starting cART but the researchers found no evidence for an association between the primary outcome and the pre-cART CD4 slope or between survival and this slope. In addition, the rate of CD4 cell count decline was not significantly associated with progression to AIDS or death among 1731 HIV-positive, symptom-free patients with CD4 cell counts above 350 cells/µl who were studied before cART was developed.

**What Do These Findings Mean?:**

These findings suggest that knowledge of the rate of CD4 cell count decline will not improve the prediction of clinical outcome in HIV-positive patients with a CD4 cell count above 350 cells/µl. Indeed, the findings show that the rate of CD4 cell decline in individual patients is highly variable over time. Consequently, a rate measured at one time cannot be used to reliably predict a patient's future CD4 cell count. Because this was an observational study, patients with the greatest rate of decline in their CD4 cell count might have received better care than other patients, a possibility that would lessen the effect of the rate of CD4 cell count decline on outcomes. Nevertheless, the findings of this study strongly suggest that knowledge of the current CD4 cell count and an assessment of other established risk factors for progression to AIDS are sufficient when deciding whether to initiate cART in symptom-free HIV-positive patients.

**Additional Information:**

Please access these Web sites via the online version of this summary at http://dx.doi.org/10.1371/journal.pmed.1000239.

- Information is available from the US National Institute of Allergy and Infectious Diseases on HIV infection and AIDS
- HIV InSite has comprehensive information on all aspects of HIV/AIDS, including information on treatments and treatment guidelines
- Information is available from Avert, an international AIDS charity, on all aspects of HIV/AIDS, including information on treatments for HIV and AIDS, when to start treatment, and the stages of HIV infection (in English and Spanish)
- Information on CASCADE is available

## Introduction

CD4 cell count is a strong predictor of the subsequent risk of AIDS or death in both untreated HIV-infected individuals and in those initiating combination antiretroviral therapy (cART) [1]–[5]. It is, therefore, a key marker for clinicians when deciding whether to initiate cART in asymptomatic patients. Current guidelines recommend treatment of asymptomatic patients before [6] or when their CD4 cell count drops below 350 cells/µl [7],[8]. The US Department of Health and Human Services (DHHS) panel also gives a moderate to strong recommendation to initiate cART in patients with a CD4 cell count between 350 and 500 cells/µl and is divided on whether to favour cART initiation in patients with CD4 cell count >500 cells/µl [7]. In addition, the International AIDS Society-USA (IAS-USA) guidelines suggest clinicians should consider cART initiation given a rapid CD4 cell count decline (>100 cells/µl per year) regardless of CD4 cell count [6], and the European AIDS Clinical Society (EACS) guidelines suggest cART should be considered given a CD4 cell count decline >50–100 cells/µl per year in patients with 350–500 CD4 cells/µl [8].

In the absence of evidence from randomised controlled trials, large collaborative cohort studies have used different modelling approaches to estimate the effect of initiating, rather than deferring, cART at different CD4 cell levels. The most recent data from cohort studies indicate that initiating cART at a CD4 cell count above 350 cells/µl may reduce the risk of AIDS and death [9],[10]. However, it is not known whether the dynamics of CD4 cell depletion, represented by the pre-cART CD4 cell slope, carry additional prognostic value for outcomes after cART initiation. Of note, in untreated patients the rate of CD4 cell decline explains only 3% and 7% of the variability in the time to AIDS and death respectively, and there is only a weak correlation between an initial viral load and the subsequent rate of CD4 cell decline [1],[11].

We consider whether CD4 cell decline is related to prognosis in patients initiating cART as well as those not treated with cART, and whether it is, therefore, relevant to the decision on whether to initiate therapy in asymptomatic patients. We use data from CASCADE (Concerted Action on SeroConversion to AIDS and Death in Europe), a large collaborative cohort study of individuals with documented evidence of the date of their HIV seroconversion [12],[13].

## Methods

### Patients and Procedures

CASCADE is a collaboration of 23 cohorts: 20 cohorts from Europe, two cohorts from Australia, and one cohort from Canada [13]. All cohorts received approval from their individual ethics review boards except for the Danish cohort, which received approval from the National Data Registry Surveillance Agency (because Danish law allowed collection and pooling of anonymised clinical data with approval from this agency alone). Two ethics review boards deemed their cohort participants exempt from providing signed informed consent. Signed informed consent was obtained from all others. Approval was also given by all ethics review boards to pool anonymised data for analyses and dissemination.

Each cohort provides data on HIV-1 infected individuals with a well-known date of HIV seroconversion. Our analysis was based on the 2008 update of CASCADE data. For most (88%) of the 19,615 patients in this update, the date of HIV seroconversion was estimated as the midpoint between the first positive HIV antibody test and the last documented negative HIV antibody test result. The time difference between the first positive and last negative HIV antibody test was less than 3 y in all cases, and less than 2 y or 1 y for 87% and 61% of such estimates, respectively. For the remaining patients, the date of seroconversion was based either on laboratory evidence of seroconversion (9%)—real-time PCR positivity in the absence of HIV antibodies or antigen positivity with fewer than four bands on a Western blot; on the date of a seroconversion illness (and an earlier documented negative HIV test at most 3 y apart) (2%); or the most likely date of an infected factor VIII concentrate infusion for haemophiliacs (1%).

Our main study population comprised all treatment-naïve individuals who started cART in 1996 or later, aged at least 16 y at the time of seroconversion, with a viral load and CD4 cell count measured either at cART initiation or within 91 d prior to initiation, and with at least one earlier CD4 cell count (and an interval between the two measurements of at least 3 mo). cART was defined as either a single or boosted protease inhibitor (PI)-based regimen or a non-nucleoside reverse transcriptase inhibitor (NNRTI)-based regimen in combination with at least two nucleoside or nucleotide reverse transcriptase inhibitors (NRTIs), or a triple NRTI regimen.

Our primary endpoint was the time from cART initiation to a first new AIDS event or death. Reoccurrence of previous opportunistic infections were not deemed to be new AIDS events as it would be difficult to distinguish an ongoing disease from a relapse [14]. Patients without an event were censored at the date of their last clinic visit. As a secondary endpoint, we analysed overall survival.

In a supplementary analysis we included patients not treated with cART during the period 1989 to 1995 (i.e., before the introduction of cART) [9]. The study population for this supplementary analysis comprised all patients with a recorded CD4 cell count in the year 1993, with no prior AIDS event and with at least one prior CD4 cell count recorded between 1989 and 1993. Baseline for the survival analysis was the date of the patient's last recorded CD4 cell count in 1993 and the time-to-event endpoint was defined as the time from baseline until AIDS or death. Patients without an AIDS event or death before December 31, 1995 were censored at this date (or at their last clinical visit if that visit was before this date). The supplementary analysis included patients receiving mono- and dual-antiretroviral therapy. In a sensitivity analysis, we excluded patients who initiated any antiretroviral therapy in 1993 or earlier and censored the time-to-event endpoint at the time of initiation of antiretroviral therapy if therapy was initiated in 1994 or 1995.

### Statistical Methods

We investigated whether the rate of CD4 cell decline prior to cART initiation (the “pre-cART CD4 cell slope”) is a prognostic factor for a new AIDS event or death. Univariate and adjusted effects were estimated using Cox proportional hazards models. For the latter, we adjusted for established predictors from the ART Cohort Collaboration risk model [2]–[4]: CD4 cell count, viral load (log-transformed) and age at cART initiation, a previous AIDS event, and HIV transmission through injection drug use. Adjustment for CD4 cell count (categorized as <100, 100 to <200, 200 to <350 and ≥350 cells/µl) and transmission through injection drug use was by stratification. We assessed the discrimination of 5-y predictions from these Cox models using the c index [15]. The analysis was performed both for all patients satisfying our inclusion criteria as well as for the subgroup of patients with a CD4 cell count at cART initiation >350 cells/µl.

Pre-cART CD4 cell slopes were estimated as best linear unbiased predictions using all available CD4 cell measurements from seroconversion until cART initiation and a linear mixed model with a patient-specific random intercept and a random slope for time since seroconversion. In a series of sensitivity analyses, we considered alternative estimates of CD4 cell slope using three different mixed models, a joint model for both CD4 cell count and time to cART initiation [16], and separate least squares estimates for each patient. Of the three different mixed models, the first excluded CD4 cell counts less than 6 mo following the first positive HIV test; the second included only CD4 cell counts within 2 y before cART initiation; and the third included only patients who seroconverted from 1996 onwards. The joint model allowed for the possibility of informative censoring if low pre-cART CD4 cell counts were often missing because individuals with low counts typically initiate cART. By jointly modelling longitudinal counts with a mixed model and time to cART initiation with a survival model, the survival component of this joint model acted as a missing data mechanism. The survival component of our joint model included covariates for both the date of seroconversion and cohort to account for varying treatment guidelines over time and between cohorts.

The proportion of patients with a CD4 decline of ≥100 cells/µl per year was calculated based on slope estimates from a linear mixed model or least squares. These estimates were compared to a simple calculation of a patients' CD4 cell slope at cART initiation using linear interpolation between the last two CD4 cell counts that were at least 6 mo apart.

In addition, we examined whether the assumption of a constant patient-specific pre-cART CD4 slope was supported by the data by fitting a more general model for CD4 trajectories with a random (patient-specific) intercept, a fixed (population) slope, an integrated Ornstein-Uhlenbeck process, and measurement error [17],[18]. This model has a parameter α that measures how stable within-patient slopes are over time. When α is zero, the model is equivalent to a random effects model with a constant patient-specific CD4 slope; when α takes a large (positive) value, the model implies that patient-specific CD4 trajectories are highly variable over time, randomly fluctuating around a population slope according to a Brownian motion process. This model was found to better fit our data than alternative nonlinear models of CD4 cell decline (see Text S1).

All formal model comparisons were based on square-root–transformed CD4 cell counts as this improved the fit of all models. However, we report CD4 cell slope estimates and effect measures of the CD4 cell slope on outcome on the basis of models of untransformed data as rates of CD4 cell decline on the original scale are much easier to interpret than estimates on the square-root scale. Analyses on the original and the square-root scale lead to highly correlated CD4 cell slope estimates and to very similar conclusions regarding the influence of CD4 cell slope on outcomes in Cox regression analyses. As an example, slope estimates based on the primary mixed effects model with and without prior square-root transformation showed a Spearman rank correlation of 0.94.

In the supplementary analysis of data from the pre-cART era, CD4 slopes were estimated as for the main analysis (i.e., with a linear mixed effects model with a random intercept and slope). The Cox regression model for the time to AIDS or death was adjusted for the CD4 cell count and age at baseline, and HIV transmission through injection drug use. Adjustment for CD4 cell count and HIV transmission was by stratification as for the primary analysis. We could not adjust for HIV viral load as this was seldom measured in the pre-cART era.

All analyses were carried out with the statistical software R version 2.8.0 [19]; the contributed R package JM was used for joint modelling [20].

## Results

Of 6,603 treatment-naïve patients initiating cART since 1996 and aged at least 16 y at seroconversion, 2,820 patients fulfilled our inclusion criteria. Patients were excluded because they had fewer than two recorded CD4 cell counts prior to cART initiation (*n = *3,177) or were lacking a CD4 cell count (*n = *54), a viral load measurement (*n = *303), or both (*n = *249) within 91 d prior to cART initiation. Among those included, the median time from seroconversion to initiating cART was 3.5 y; cART was initiated at a median age of 36 y and with a median CD4 cell count of 289 cells/µl (Table 1). Excluded patients initiated cART earlier (median of 0.8 y from seroconversion to initiating cART), at a higher CD4 cell count (median 360 cells/µl) or were treated for seroconversion illness.

**Table 1:** Characteristics of 2,820 treatment-naïve patients from the CASCADE collaboration initiating cART in 1996 or later.

Characteristic	Summary Statistic: *n* (%) or Median (Interquartile Range)			
	All Patients (*n* = 2,820)		Patients with >350 CD4 Cells/µl at cART Initiation (*n* = 961)	
**Female**	578	(20%)	215	(22%)
**Age at cART initiation (y)**	36	31–43	34	30–41
**Most likely source of infection**				
Sex between men and women	734	(26%)	249	(26%)
Sex between men and men	1,669	(59%)	545	(57%)
Injecting drug use	247	(9%)	93	(10%)
Other/unknown	170	(6%)	74	(8%)
**Type of first cART**				
Boosted PI	705	(25%)	171	(18%)
Single PI	680	(24%)	358	(37%)
NNRTI	1,166	(41%)	316	(33%)
Triple NRTI	269	(10%)	116	(12%)
**Prior AIDS**	216	(8%)	50	(5%)
**Time between last negative and first positive HIV test (y)**	0.82	0.35–1.49	0.78	0.38–1.41
**Time from seroconversion** a **to cART initiation (y)**	3.52	1.86–6.41	3.11	1.63–5.84
**Viral load (log10 copies/ml) at cART initiation**	4.78	4.11–5.25	3.66	4.54–5.05
**CD4 cell count (cells/µl) at cART initiation**	289	206–398	468	393–580
**Number of CD4 measurements before cART initiation**	7	4–12	6	4–10
**Estimated pre-cART CD4 slope (cells/µl per year)** b	−61	−81 to –46	−58	−74 to –37

a Midpoint between last negative and first positive test for 2,588 patients; date of laboratory evidence of seroconversion or seroconversion illness for the remaining patients.

b Predictions from a linear mixed model with a random intercept and a random slope for the covariate time since seroconversion.

PI, protease inhibitor; NNRTI, non-nucleoside reverse transcriptase inhibitor; NRTI, reverse transcriptase inhibitor.

Estimates from the linear mixed effects model gave a median (interquartile range [IQR]) pre-cART CD4 cell decline of 61 (46–81) cells/µl per year and 13% of individuals had a decline >100 cells/µl per year. Separate least squares estimates for each patient gave a median pre-cART CD4 decline of 74 (31–145) cells/µl per year and 38% of individuals had a decline >100 cells/µl per year. Finally, if slopes were estimated by interpolating the last two CD4 measurements at least 6 mo apart before cART initiation, median (IQR) decline was 114 (32–229) cells/µl per year and 54% of individuals had a decline >100 cells/µl per year.

During 10,296 person-years of follow-up (with a median follow-up of 3.2 y per patient), 255 patients experienced the primary endpoint of a new AIDS event or death (with 92 deaths). When cross-classified by the quartiles of pre-cART CD4 slope, these 255 events were partitioned into 71, 60, 56, and 68 events respectively. A total of 125 patients died during follow-up.

In an unadjusted Cox model, the hazard ratio for AIDS or death was 0.99 (95% confidence interval [CI] 0.95–1.02) for each 10 cells/µl per year reduction in pre-cART CD4 cell decline (Table 2). The discrimination of this variable (the c index) was 0.52; lower than any individual component in the ART Cohort Collaboration risk prediction model (with c indices ranging from 0.56 for age to 0.64 for CD4 cell count). In an adjusted Cox model, the hazard ratio for AIDS or death was 1.01 (95% CI 0.97–1.04) for each 10 cells/µl per year reduction in pre-cART CD4 cell decline, and multivariate Cox models with and without pre-cART CD4 slope had identical c indices of 0.70. There was also no association between pre-cART CD4 slope and survival (Table 2). Sensitivity analyses using alternative estimates of pre-cART CD4 cell slope gave consistent results in adjusted Cox models for the time to a new AIDS event or death, with *p*-values ranging from 0.24 to 0.88. There was also no evidence of an association of pre-cART CD4 slope and progression to AIDS or death when we restricted our analysis to 961 patients with a CD4 cell count >350 cells/µl at cART initiation (Table 2) or when the pre-cART CD4 slope was entered as a binary covariate (≥100 versus <100 cells/µl decline per year) instead of as a continuous covariate (unpublished data).

**Table 2:** Prognostic strength of the pre-cART CD4 slope for the prediction of clinical outcomes in 2,820 treatment-naïve patients from the CASCADE collaboration initiating cART in 1996 or later.

Outcome and Type of Summary	All Patients (*n* = 2,820)		Patients with >350 CD4 cells/µl at cART Initiation (*n* = 961)	
**Outcome: time to new AIDS event or death**				
Number of events	255	(9%)	9	(9%)
Event rate per 100 patient-years of follow-up (95% CI)	2.48	(2.17–2.78)	2.04	(1.61–2.46)
Unadjusted effect of pre-cART CD4 slope on outcome				
HR for +10 cells/µl per year higher slope (95% CI)	0.99	(0.95–1.02); *p* = 0.39	0.99	(0.94–1.05); *p = *0.85
Discrimination (c index)	0.52	—	0.55	—
Adjusted effect of pre-cART CD4 slope^a^				
HR for +10 cells/µl per year higher slope (95% CI)	1.01	(0.97–1.04); *p = *0.65	1.02	(0.96–1.08); *p = *0.48
Discrimination (c index) of model without CD4 slope	0.70	—	0.65	—
Discrimination (c index) of model with CD4 slope	0.70	—	0.65	—
**Outcome: time to death**				
Number of events	125	(4%)	47	(5%)
Event rate per 100 patient-years of follow-up (95% CI)	1.12	(0.93–1.32)	1.00	(0.71–1.28)
Unadjusted effect of pre-cART CD4 slope on outcome				
HR for +10 cells/µl per year higher slope (95% CI)	1.01	(0.96–1.06); *p = *0.83	0.97	(0.90–1.05); *p = *0.41
Discrimination (c index)	0.50		0.62	
Adjusted effect of pre-cART CD4 slope^a^				
HR for +10 cells/µl per year higher slope (95% CI)	1.01	(0.96, 1.06); *p = *0.65	0.98	(0.91–1.05); *p = *0.52
Discrimination (c index) of model without CD4 slope	0.73	—	0.69	—
Discrimination (c index) of model with CD4 slope	0.73	—	0.72	—

The pre-cART CD4 slope was estimated by a linear mixed model with a random intercept and a random slope for time since seroconversion.

a Adjusted for established predictors from the ART Cohort Collaboration risk model: CD4 cell count, viral load (log-transformed) and age at cART initiation, a previous AIDS event, and HIV transmission through injection drug use.

HR, hazard ratio.

Nonlinear models for pre-cART CD4 cell decline fitted the data significantly better than the linear models discussed above and the model based on an integrated Ornstein-Uhlenbeck process led to the best fit (see Text S1). This model gave a 95% CI for the parameter α from 36 to infinity, which corresponds to highly variable within-patient rates of CD4 cell decline over time, so that the correlation between a patient's current CD4 cell slope and their CD4 cell slope in 6 mo time is estimated to be essentially zero [18].

A total of 3,078 patients were included in the supplementary analysis using data from the pre-cART era. Baseline characteristics are displayed in Table 3. There was a significant association between CD4 cell slope and the 2-y risk of AIDS events or deaths in these cART-naive patients with an adjusted hazard ratio of 0.96 (95% CI 0.94–0.98) for each 10 cells/µl per year reduction in CD4 cell decline. However, the CD4 cell slope did not significantly affect the progression to AIDS or death when the analysis was restricted to 1,731 patients with CD4 >350 cells/µl at baseline (hazard ratio 0.99, 95% CI 0.94–1.03, for each 10 cells/µl per year reduction in CD4 cell decline; Table 4). By contrast, CD4 cell count and age remained highly significant predictors in this subgroup. The sensitivity analysis that excluded all patients receiving mono- or dual-antiretroviral therapy was based on 1,797 patients with a median CD4 cell count of 480 cells/µl at baseline, a median CD4 cell slope of −47 cells/µl per year, and 137 subsequent AIDS events or deaths. The adjusted hazard ratio of the CD4 cell slopes on AIDS events or deaths was 0.97 (95% CI 0.93–1.01; *p = *0.15) for each 10 cells/µl per year reduction in CD4 cell decline if patients were included regardless of CD4 cell count and 0.98 (95% CI 0.92–1.04; *p = *0.48) in 1,346 patients with CD4 cell count >350 cells/µl at baseline.

**Table 3:** Characteristics of 3,078 AIDS-free patients from the CASCADE collaboration with a CD4 cell count in 1993 and at least one prior CD4 cell count.

Characteristic	Summary Statistic - *n* (%) or Median (Interquartile Range)			
	All Patients (*n = *3,078)		Patients with >350 CD4 cells/µl at Baseline (*n = *1,731)	
**Female**	677	(22%)	411	(24%)
**Age at baseline** a **(y)**	32	28–38	31	27–37
**Most likely source of infection**				
Sex between men and women	567	(18%)	325	(19%)
Sex between men and men	1,570	(51%)	899	(52%)
Injecting drug use	550	(18%)	310	(18%)
Other/unknown	391	(13%)	197	(11%)
**Time between last negative and first positive HIV test (y)**	0.65	0.27–1.17	0.66	0.33–1.17
**Time from seroconversion** b **to baseline** a **(y)**	3.96	2.34–5.98	3.43	2.03–5.35
**CD4 cell count (cells/µl) at baseline** a	390	252–550	524	432–696
**Number of CD4 measurements between 1989 and baseline** a	7	4–11	5	3–9
**Estimated CD4 slope (cells/µl per year)** c **at baseline** a	−46	−70 to −26	−44	−68 to −22

a Baseline refers to the date of the patient's last CD4 cell count recorded in 1993.

b Midpoint between last negative and first positive test for 2,773 patients; date of laboratory evidence of seroconversion or seroconversion illness for the remaining patients.

c Predictions from a linear mixed model with a random intercept and a random slope for the covariate time since seroconversion.

**Table 4:** Prognostic strength of CD4 slope for the prediction of clinical outcomes occurring between 1994 and 1995 in 3,078 AIDS-free patients from the CASCADE collaboration with a CD4 cell count in 1993 and at least one prior CD4 cell count.

Outcome and Type of Summary	All Patients (*n = *3,078)		Patients with >350 CD4 Cells/µl at Baseline (*n = *1,731)	
**Outcome: time to AIDS event or death**				
Number of events	549	(18%)	115	(7%)
Event rate per 100 patient-years of follow-up (95% CI)	10.19	(9.34–11.04)	3.57	(2.92–4.23)
Unadjusted effect of CD4 slope on outcome				
HR for +10 cells/µl per year higher slope (95% CI)	0.93	(0.91–0.95); *p<*0.001	0.99	(0.95–1.03); *p = *0.68
Discrimination (c index)	0.61	—	0.52	—
Adjusted effect of CD4 slope^a^				
HR for +10 cells/µl per year higher slope (95% CI)	0.96	(0.94–0.98); *p<*0.001	0.99	(0.94–1.03); *p = *0.55
Discrimination (c index) of model without CD4 slope	0.78	—	0.64	—
Discrimination (c index) of model with CD4 slope	0.78	—	0.64	—
**Outcome: time to death**				
Number of events	237	(8%)	33	(2%)
Event rate per 100 patient-years of follow-up (95% CI)	4.14	(3.61–4.66)	1.00	(0.66–1.35)
Unadjusted effect of CD4 slope on outcome				
HR for +10 cells/µl per year higher slope (95% CI)	0.93	(0.91–0.95); *p<*0.001	1.01	(0.94–1.10); *p = *0.74
Discrimination (c index)	0.63	—	0.52	—
Adjusted effect CD4 slope^a^				
HR for +10 cells/µl per year higher slope (95% CI)	0.99	(0.96–1.01); *p = *0.28	1.01	(0.99–1.09); *p = *0.73
Discrimination (c index) of model without CD4 slope	0.83	—	0.67	—
Discrimination (c index) of model with CD4 slope	0.83	—	0.66	—

CD4 slope was estimated by a linear mixed model using CD4 cell counts recorded between January 1, 1989, and December 31, 1993, with a random intercept and a random slope for time since seroconversion.

a Adjusted for CD4 cell count, age and HIV transmission through injection drug use.

HR, hazard ratio.

## Discussion

We found no evidence that knowledge of pre-cART CD4 cell slope improves the prediction of the risk of a new AIDS event or death in our analysis of 2,820 treatment-naïve patients who then started cART. The 95% CI for the adjusted hazard ratio of 0.97–1.04 for each 10 cells/µl per year reduction in pre-cART CD4 cell decline is sufficiently narrow to exclude a large effect.

Our study only included patients with a known date of seroconversion. In a general population of HIV-infected individuals, patients presenting with an unknown duration of infection will have even fewer CD4 cell measurements prior to initiating cART making it more difficult to estimate a reliable pre-cART CD4 cell slope for such patients [21],[22]. We estimated CD4 slopes from untransformed CD4 cell counts using a linear mixed model because this approximates how a physician might intuitively estimate the CD4 cell slope for a patient: first estimate the patient's slope by eye or using standard linear regression but then adjust that estimate towards the population slope if only few CD4 cell counts are available for that patient. We also found similar prognostic performance on the basis of alternative estimates of the CD4 cell slope.

However, the proportion of patients with a CD4 cell decline >100 cells/µl per year varied from 13% to 54%, depending on the method used to estimate a patient's CD4 cell slope. The variability of slope estimates, and hence the prevalence of patients with a rapid CD4 cell decline, decreased markedly as more information was included in the calculation of a patient's slope. Only two CD4 cell counts (the interpolation of the last two measurements) gave highly variable slope estimates and a high percentage of patients with a rapid decline, using all measurements for each patient (least-squares estimation) was intermediate, while adding background information on the distribution of slopes in the general population (mixed model estimation) gave the least variable slope estimates and the lowest percentage of patients with a rapid decline. Current treatment guidelines give explicit thresholds for CD4 cell count decline, from 50 to 100 cells/µl per year [6],[8], such that cART initiation should be considered should these thresholds be exceeded, but guidelines do not say how the CD4 cell slope should be measured.

There are two factors that contribute to the poor prognostic performance of the CD4 slope. First, CD4 cell slopes are often imprecisely estimated because only few CD4 cell counts are available per patient [1]. Second, we found strong evidence that the rate of CD4 decline over time is nonlinear. Indeed, our more general model based on an integrated Ornstein-Uhlenbeck process suggests that the very concept of a constant within-patient CD4 cell decline prior to treatment is probably flawed. It would seem that a better description of CD4 cell decline is, for each patient, a highly variable decline over time fluctuating randomly around a population slope. This conclusion is consistent with earlier studies of untreated patients from the Multicenter AIDS Cohort Study [17],[18]. The fact that CD4 cell decline is not constant for each patient but highly variable would explain why pre-cART CD4 cell slope has so little prognostic value.

A number of studies have shown an association between CD4 cell slope with clinical outcome in the pre-cART era [1],[23]. The reported effects of CD4 cell slope in cART-naïve patients were significant but small—for example, an adjusted HR for progression to AIDS of 1.02 for each 10 cells/µl per year decline in CD4 cell slope [23]. We confirm the existence of such a small effect in our supplementary analysis of 3,078 patients from the pre-cART era but note that the effect did not reach statistical significance in patients with a CD4 cell count >350 cells/µl, the patient subgroup for whom a rapid CD4 decline is considered relevant under current guidelines. This result could reflect that it is generally more difficult to predict AIDS events or deaths in a lower-risk population of patients with a high CD4 cell count; indeed, the prognostic ability of the absolute CD4 cell count also decreased in this subpopulation (but remained highly significant).

The idea that the rate of CD4 cell decline is informative seems to have its origins in studies showing that AIDS is often preceded by a sudden fall in CD4 cell count [24]. Such studies led some to conclude that differences in AIDS progression rates can largely be explained by differences in rates of CD4 cell decline [25]. However the reverse is not necessarily true—sudden falls in CD4 cell count are not always a precursor to AIDS. This was shown in untreated patients in the early 1990s: “A more rapid long term decline in CD4 count can be seen retrospectively to have occurred in patients who progress to AIDS, but at any point before the development of AIDS the near term variability precludes assigning any prognostic significance to precipitous changes other than that associated with a low CD4 count.” [24]. This message appears to have been lost and current guidelines imply that clinicians should consider cART for patients with a CD4 cell count above 350 cells/µl where there has been a rapid decline in CD4 cells [6],[8].

It is important to note that our study addresses a prognostic and not a causal research question. To be specific, assume two asymptomatic treatment-naïve HIV-positive patients, both with an identical CD4 cell count >350 cell/µl and identical other risk factors (viral load, age, etc.) at the current assessment, but a different earlier CD4 dynamics, i.e., one with a steep CD4 decline and the other with a more shallow decline. Our study assesses whether these patients have a different prognosis. We find that the CD4 slope is not predictive of future AIDS events or deaths if cART is initiated immediately, i.e., both patients are expected to have a similar outcome in this situation. Similarly, if cART is deferred in both patients, we find no evidence that they have a different risk of progression to AIDS while untreated. Because of the lack of prognostic information of the CD4 cell slope in both situations, it seems rational to ignore the CD4 slope when making clinical decisions and, in particular, when deciding whether to initiate cART or not.

Recent publications from cohort studies addressed the causal question of the optimal CD4 threshold for cART initiation by mimicking a hypothetical randomized clinical trial in their analyses and thus avoided bias that occurs if deaths and AIDS events prior to cART initiation or lead time are ignored [9],[10]. Since it is not possible to randomize patients to one CD4 slope or another, a randomized clinical trial for our research question is not possible. However, with a randomized trial one could, in principle, evaluate explicitly whether patients with rapid CD4 progression benefit from immediate cART treatment. To validate current treatment guidelines related to the CD4 slope, such a trial would compare cART initiation at a CD4 cell count of 350 cell/µl, regardless of the rate of CD4 decline, versus cART initiation at a CD4 cell count of 350 cells/µl combined with earlier cART initiation in patients with a steep rate of decline. It is a limitation of our study, that we address this causal research question only indirectly.

Importantly, however, such a trial would not answer the question of whether the CD4 cell slope is relevant for prognosis and medical decision making in general. Indeed, the trial may well show the superiority of the latter treatment arm because cART would be initiated at a higher CD4 cell count in a subgroup (i.e., patients with a steep decline) and earlier initiation of cART may be beneficial regardless of the CD4 slope [10].

A further limitation of our study is that it is based on observational cohort data and not experimental data. Although it is unlikely, we can not rule out that patients with a steeper rate of CD4 decline received better care than other patients and so had a similar outcome to other patients. In addition, we include only patients with a known date of seroconversion and our study population may not be representative for the general population of HIV-infected individuals. Second, it is statistically impossible to conclude that a marker such as the CD4 slope has no effect on clinical outcome. Our analyses were based on relatively large sample sizes and CIs for the effect of the CD4 slope were sufficiently narrow to rule out a large effect. However, in the subgroup of patients with CD4 >350 cells/µl, the number of subsequent deaths was relatively low. Third, our supplementary analysis is based on data from the pre-cART era, i.e., 1989–1995. We are thus implicitly assuming that current patients would have the same prognosis as patients from the pre-cART era if cART was not initiated. Finally, we were unable to adjust for HIV viral load in the supplementary analysis as the assays were not generally available in the pre-cART era. However additional adjustment for HIV-viral load would be expected to further diminish the effect of the CD4 slope as HIV-viral load and CD4 slope are correlated [1],[11].

In conclusion, our results suggest that the prognosis of patients with a CD4 cell count >350 cells/µl is largely unaffected by the dynamics by which they arrived at a given CD4 cell count both if cART is initiated or if cART is withheld. Knowledge of the current CD4 cell count and an assessment of established risk factors are sufficient when deciding whether to initiate cART in asymptomatic HIV patients. The high variability of within-patient CD4 slopes over time show that prior rates of CD4 decline cannot be used to reliably predict a patient's future CD4 cell count and thus guide the CD4 cell monitoring frequency prior to cART initiation. The implication for monitoring patients prior to starting therapy is therefore that CD4 cell counts should be measured regularly according to current guidelines (i.e., at least every 6 mo) [7],[8], something that may be difficult to achieve in a resource-limited setting [26].

## Supporting Information

Text S1 Technical appendix: nonlinear models for CD4 cell decline.
(0.04 MB DOC)

## Abbreviations

cART: combination antiretroviral therapy, CASCADE, Concerted Action on SeroConversion to AIDS and Death in Europe
