# Supplementary material for: Pretreatment CD4 Cell Slope and Progression to AIDS or Death in HIV-Infected Patients Initiating Antiretroviral Therapy—The CASCADE Collaboration: A Collaboration of 23 Cohort Studies
Source: PLoS Med. 2010 Feb 23;7(2):e1000239. doi: 10.1371/journal.pmed.1000239 (PMC2826377; doi:10.1371/journal.pmed.1000239)
Supplement: Text S1 — Technical appendix: nonlinear models for CD4 cell decline. (0.04 MB DOC) [file pmed.1000239.s001.doc]

**Text S1**

**Technical appendix: Nonlinear models for CD4 cell decline**

**Methods**

The following models were evaluated:

1. LINEAR: A linear mixed effects model with a random intercept and a random slope for the time since seroconversion (the model used in the text to estimate the CD4 slope).
2. QUADRATIC: A mixed effects model with a quadratic time trend, at both the population and patient level (i.e. fixed and random effects for the intercept, linear term, and quadratic term).
3. CUBIC: A mixed effects model with a cubic time trend, at both the population and patient level.
4. PIECEWISE LINEAR: A mixed effects model with a piecewise linear trend, with knots at 2, 4, 6, and 8 years after seroconversion (at both the population and patient level).
5. ORNSTEIN-UHLENBECK: A model with a random intercept, a fixed (population) slope and an integrated Ornstein-Uhlenbeck process. [17,18]
6. BROWNIAN MOTION: A model with a random intercept, a fixed (population) slope and a Brownian motion process. [17,18] This model is a special case of Model 5. It corresponds to the case where parameter α is infinite in the integrated Ornstein-Uhlenbeck process (i.e. the patient’s slope is highly variable over time) so that the CD4 trajectory fluctuates randomly around a population slope according to a Brownian motion process.

All models were fit to square-root transformed CD4 cell counts and time zero was the date of seroconversion. The models were compared in terms of the Akaike information criterion (AIC); a lower value of AIC indicates a better model fit.

The models were evaluated on the following datasets:

- DATASET 1: CD4 measurements from seroconversion to cART initiation in 2820 treatment-naïve patients initiating cART since 1996 (the data used for the primary analysis in this study).
- DATASET 2: The above data restricted to the 1348 patients contributing at least 8 CD4 cell counts before cART initiation.
- DATASET 3: CD4 measurements from 1989-1993 in 3078 AIDS-free patients with a CD4 cell count in 1993 and at least one prior CD4 cell count (the data used for the supplementary analysis in this study).

All analyses were carried out with the statistical software R version 2.9.1 (R Foundation for Statistical Computing, Vienna, Austria); mixed effects models were fitted with the R-package lme4.

Results

| Akaike information criterion (AIC) relative to a linear model (Model 1) | | | | |
| --- | --- | --- | --- | --- |
|  | Number of model parameters | DATASET | | |
| Model |  | 1 | 2 | 3 |
| 1. LINEAR | 6 | 0 | 0 | 0 |
| 2. QUADRATIC | 10 | -1427 | -1195 | -477 |
| 3. CUBIC | 15 | -1979 | -1634 | -897 |
| 4. PIECEWISE LINEAR | 28 | -2570 | -2119 | -1161 |
| 5. ORNSTEIN-UHLENBECK | 6 | -3064 | -2602 | -1363 |
| 6. BROWNIAN MOTION | 5 | -3066 | -2604 | -1365 |

**Conclusions**

All non-linear models have substantially lower AIC than the linear model and thus fit the data better. This is strong evidence for a non-linear CD4 cell decline. However, unlike the linear model, these more complicated models lack an easily interpretable patient summary measure such as a slope.

Amongst all models, Model 5 based on Brownian motion (corresponding to an integrated Ornstein-Uhlenbeck process with α=∞) fits all three datasets best and is the most parsimonious in terms of the number of model parameters. This is consistent with previous comparisons,[17,18] and implies that the rate of CD4 cell decline is highly variable over time.
